# An Agent-Based Model of Cellular Dynamics and Circadian Variability in Human Endotoxemia

**DOI:** 10.1371/journal.pone.0055550

**Published:** 2013-01-30

**Authors:** Tung T. Nguyen, Steve E. Calvano, Stephen F. Lowry, Ioannis P. Androulakis

**Affiliations:** 1 BioMaPS Institute for Quantitative Biology, Rutgers University, Piscataway, New Jersey, United States of America; 2 Department of Biomedical Engineering, Rutgers University, Piscataway, New Jersey, United States of America; 3 Department of Surgery, Robert Wood Johnson Medical School, UMDNJ, New Brunswick, New Jersey, United States of America; Albert Einstein College of Medicine, United States of America

## Abstract

As cellular variability and circadian rhythmicity play critical roles in immune and inflammatory responses, we present in this study an agent-based model of human endotoxemia to examine the interplay between circadian controls, cellular variability and stochastic dynamics of inflammatory cytokines. The model is qualitatively validated by its ability to reproduce circadian dynamics of inflammatory mediators and critical inflammatory responses after endotoxin administration *in vivo*. Novel computational concepts are proposed to characterize the cellular variability and synchronization of inflammatory cytokines in a population of heterogeneous leukocytes. Our results suggest that there is a decrease in cell-to-cell variability of inflammatory cytokines while their synchronization is increased after endotoxin challenge. Model parameters that are responsible for IκB production stimulated by NFκB activation and for the production of anti-inflammatory cytokines have large impacts on system behaviors. Additionally, examining time-dependent systemic responses revealed that the system is least vulnerable to endotoxin in the early morning and most vulnerable around midnight. Although much remains to be explored, proposed computational concepts and the model we have pioneered will provide important insights for future investigations and extensions, especially for single-cell studies to discover how cellular variability contributes to clinical implications.

## Introduction

Systemic inflammation is evoked by many stimuli, including infection, trauma, invasive surgery and biological stressors in general; also, it is typically observed in critical illness [1]. While the host inflammatory response is essential to resolve an infection or repair damage to restore homeostasis, it also plays a central pathogenic role in a wide spectrum of diseases [2]. Under normal circumstances, the inflammatory response is activated, initializes a repair process and then abates [3]. However when anti-inflammatory processes fail, an amplified pro-inflammatory signal can turn what is normally a beneficial reparative process into a detrimental physiological state of severe, uncontrolled systemic inflammation [4]. Therefore, to gain a better understanding of the molecular mechanisms and physiological significance associated with inflammatory responses, alternative clinically relevant models have been proposed, including the human endotoxemia model. In this model, an intravenous administration of *E.coli* endotoxin (lipopolysaccharide) is given to healthy human subjects [5], [6]. Lipopolysaccharide (LPS), a component of the outer cell membrane of gram-negative bacteria [7], induces its injurious effects by a non-cytotoxic interaction with CD14-bearing inflammatory cells, such as macrophage-monocytes, circulating neutrophils and lung epithelial cells. These effector cells are activated through a family of Toll-like receptors (TLR) and subsequently release a network of inflammatory products. While we do not argue that the human endotoxin challenge model can precisely replicate an acute infectious or sepsis condition, we believe that human endotoxin challenge does serve as a useful model of TLR4 agonist-induced systemic inflammation while at the same time providing a reproducible experimental platform.

The inflammatory response is a complex non-linear process involving a multi-scale cascade of events mediated by a large array of immune cells and inflammatory cytokines [8]. At the cellular level, innate immune cells are activated resulting in the production and release of pro-inflammatory and anti-inflammatory cytokines to the systemic circulation for cell communication [9], [10]. Anti-inflammatory cytokines counteract the effects of pro-inflammatory cytokines and the relative concentration or balance between them strongly affects to the degree and extent of the response [11], [12]. At a higher level, the hypothalamic-pituitary-adrenal (HPA) axis and the sympathetic nervous system (SNS) produces stress hormones [13] whose pattern of release follow broad circadian rhythmicity which plays critical roles in immune responses [14], [15], [16], [17]. This rhythmicity is regulated by the 24 hour light/dark cycle, exerting diurnal effects on numerous inflammatory cytokines [18], [19]. The complexity of the overall response has encouraged the development of mathematical and computational models as a means of exploring the connections between multiple components.

Various modeling approaches have been proposed, but generally they can be classified into two main categories: equation-based and agent-based modeling [20], [21], [22]. In previous studies, we developed a mathematical model of the human endotoxemia using equation-based modeling technique with ordinary differential equations (ODE) 23,24,25. However, deterministic ODE models assume homogeneity and perfect mixing within compartments, while ignoring spatial effects [21]. Given that stochasticity and heterogeneity have profound effects on the function of biological systems [26], [27], [28], agent-based modeling (ABM) – an alternative, more intuitive, approach has been explored. ABM is an object-oriented, rule-based, and discrete modeling method [29], [30] where interactions between agents (cells, molecules) are nonlinear, stochastic, spatial, and are described by asynchronous movements through multiple compartments. The usefulness and applicability of ABMs vary but some have been applied to immunological problems and findings derived from these models generated a lot of insights into the interactions and dynamics at the cellular level in immune responses. For example, Jenkins and colleagues [31] investigated B-T cell interactions in the absence of directed cell chemotaxis during the first 50 hr of a primary immune response to an antigen; Gary An and coworkers have pioneered many ABMs to evaluate the dynamics of the innate immune response, the efficacy of proposed interventions for SIRS/multiple organ failure (MOF) [32], [33], and the dynamics of the TLR4 signal transduction cascade to study LPS preconditioning and dose-dependent effects [34], [35]. Furthermore, they also developed a basic immune simulator (BIS) to qualitatively examine the interactions between innate and adaptive interactions of the immune responses to a viral infection [36]. In addition, there are a variety of successful agent-based simulators that have been constructed as frameworks for immunology/disease understanding and exploration e.g. IMMSIM [37], [38], SIMMUNE [39], CyCells [40].

In this study, we developed an ABM to investigate the cellular variability through the interactions and dynamics of inflammatory cytokines in acute inflammatory responses following endotoxin administration. The model naturally incorporates key biological features (e.g. stochasticity, heterogeneity, and discreteness) and physicochemical properties of biological molecules. While in previous studies [23], [24], [25] we focused on the possibility of modeling the transcriptional dynamics of cellular responses, we here attempt to capture stochastic variation in the transcriptional process, one of the key factors leading to phenotypic variation in addition to genetic and environmental variability [41], [42], 43,44. Because stochasticity is an inherent property of agent interactions, non-genetic cell-to-cell variability originated from stochastic variance is captured by our proposed model. Therefore, elucidating the relationship between the behaviors measured at the single-cell level and those measured in a population of cells is among the aims of our study in order to provide insight into the host inflammatory response under different external stimuli.

We first construct a homeostatic model of components involved in the response to human endotoxemia using the agent-based approach. Novel heuristics are proposed regarding parameter tuning with process trending analysis techniques and time-scale estimation by mapping *in silico* system behaviors to *in vivo* transcriptional responses. Inevitably there is a level of abstraction in the simulation when representing biological events using the indirect response modeling technique [45] and thus the model is validated through its ability to capture *in vivo* transcriptional responses and reproduce circadian rhythms. A critical contribution of our work is the assessment of cellular variability derived from stochastic variation in biological events, especially in transcriptional processes. By proposing a novel hypothetical measurement F_var_ derived from the balance distribution of pro- and anti-inflammatory mediators in the population of leukocytes, we extract information content conveyed by cell-to-cell variability. Sensitivity analysis is also performed to explore which model parameters greatly influence system behaviors. All in all, the main aim of this study is establishing a multi-scale modeling framework capable of simulating main characteristics of critical components in human endotoxemia to examine (i) the balance and distribution of inflammatory cytokines in a population of heterogeneous leukocytes and (ii) the interplay between circadian controls and endotoxin treatments through a novel quantity based on the cell-to-cell variability.

## Model Construction

### Assumptions and biological evidence

The model is principally constructed based on our previous studies [23], [24], [25]. First of all, high-dimensional transcriptional profiling data from human blood leukocytes following LPS administration are decomposed into four significant expression patterns. These patterns capture the essence of three inflammatory phases including a pro-inflammatory response (‘early-up’ & ‘middle-up’ expression pattern, P), a counter-regulatory/anti-inflammatory response (‘late-up’ expression pattern, A), and a dysregulation in leukocyte bioenergetics (‘down’ pattern, E) [46] (see Materials and methods). They define the basic elements (state variables) characterizing how leukocytes respond to endotoxemia.

A number of assumptions have been made to construct the model, namely: (1) peripheral blood leukocytes can be approximated as a community of leukocytes whose main behavior is characterized by asynchronous and stochastic activities without intra-cellular spatial localization; (2) the dynamics of the pro-inflammatory response, the counter-regulatory response, and the dysregulation in leukocyte bioenergetics can be characterized by patterns of corresponding pro-inflammatory cytokines, anti-inflammatory cytokines, and bio-energetic proteins; (3) different types of pro-inflammatory cytokines, anti-inflammatory cytokines, and bio-energetic proteins are represented by corresponding average delegators as P, A, and E, respectively, whose main behaviors are associated with asynchronous and stochastic activities. Lastly, it has been observed that after LPS challenge, many pro-inflammatory cytokines exhibit similar dynamics as is observed in their corresponding mRNA temporal profiles e.g. TNFα, IL6, IL8, etc [47]. IL10, an anti-inflammatory cytokine, shows a slight difference between its mRNA and protein temporal profiles. While mRNA levels of IL10 dropped during the first hour post-LPS and its protein levels rose very modestly, both profiles still exhibit up-regulation overall. Consequently, in this context, we hypothesize that the common dynamics of pro- and anti-inflammatory cytokines can be characterized by their average mRNA expression profiles.

Such expression dynamics of inflammatory cytokines are assumed to be mainly regulated by the activation of relevant transcription factors (TFs). Nuclear factor-kappa B (NFκB) was selected as the representative signaling controller underpinning the manifestation of transcriptional responses due to its essential role in the immune system [48], [49] and extensive prior computational analyses [50]. Furthermore, NFκB activity is primarily modulated by the activity of its kinase (IKK) and its inhibitor (IκB) through the Toll-like receptor (TLR) signaling pathway – a pivotal pathway subjected to crosstalk from other signals and pathways (e.g. JAK-STAT [51], [52]) [53], [54]. Such regulation can be characterized by the ubiquitous paradigm of a two-feedback mechanism: a positive- and a negative- feedback [54], [55], [56], [57]. Therefore, we hypothesize that the dynamics of inflammatory cytokines are mainly regulated by intra-cellular signaling cascades and transcription factors whose activities can be characterized by the paradigm of a two-feedback regulatory mechanism.

**Figure 1:**
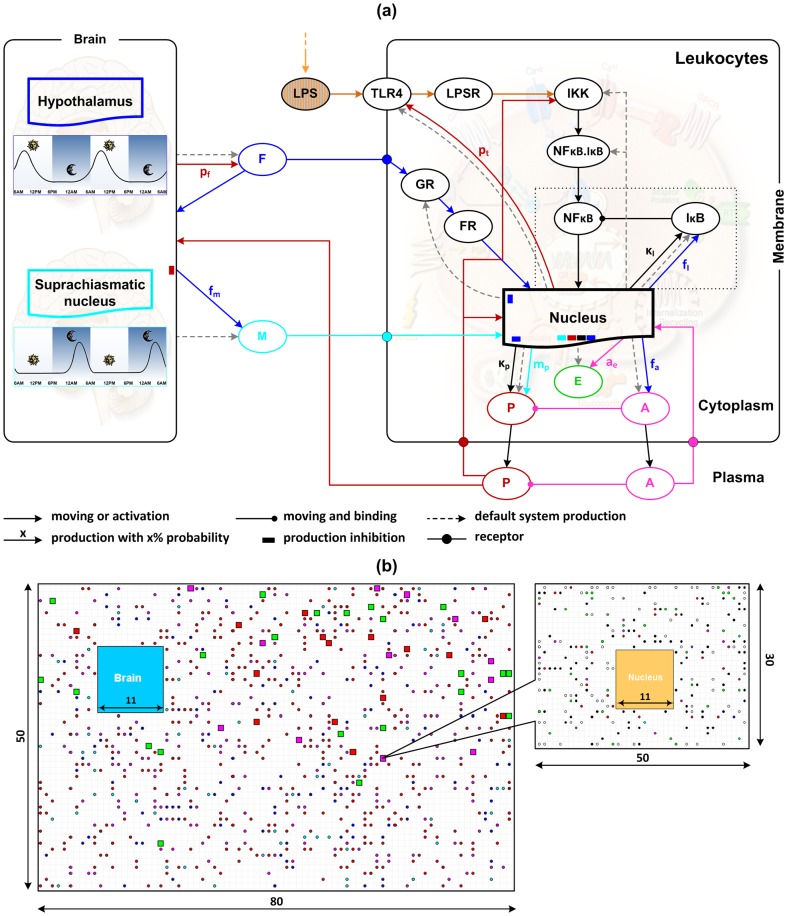
*In silico* human endotoxemia model accounting for circadian variability. (a) The rule system representation. At the cellular level, molecular interactions involve the propagation of LPS signaling on the transcriptional response level (P, A, E) through the activation of NF-kB signaling module. At the systemic level, circulating stress hormones are released from the neuro-endocrine system coupled with their circadian rhythms. The dynamics of cortisol (F) and melatonin (M) signaling from the systemic level involve molecular behaviors at the cellular level. The activities of each agent are characterized by its corresponding color. (b) A snapshot of the implemented model. Molecules are displayed with solid circles (P: red-; A: magenta-; F: blue-; M: cyan-; NFkB: yellow-; E: green-; TLR & GR: white-; IkB, IKK, NFkB.IkB: black- circles). Cells are displayed with solid squares where green squares represent for cells with an approximate number of P and A, red squares for those with the number of P greater than 1.5 fold of the number of A and magenta squares for those with A more than 1.5 fold of P.

At the systemic level, pro-inflammatory cytokines released from the innate immune system induce signals activating the hypothalamic-pituitary adrenal (HPA) axis, thus controlling the secretion of glucocorticoids (cortisol in primates or corticosterone in rodents) [58], [59]. Of particular interest is the hormone melatonin given its role as a mediator in the crosstalk between the suprachiasmatic nucleus (SCN) and the immune system [60], [61]. The corresponding hormone levels exhibit a circadian pattern with strong effects on the production of inflammatory cytokines [18], [19]. While cortisol reaches its peak in the early morning [62], melatonin’s peak production occurs late at night and remains at a low level for the rest of the day [61], [63]. Therefore, in this model cortisol (F) is hypothesized to be mainly controlled by the hypothalamus (HPT) while melatonin (M) is regulated by the SCN. The representation of the proposed model, including all components and associated interactions, is shown in **Figure 1**. This describes how the system is controlled and provides an intuitive illustration of the entire model behavior. A snapshot of the implemented model is also presented. Simulated molecular types and their corresponding characteristics are shown in **Table 1**. Details of model components, rules, and parameters are discussed as follows.

**Table 1:** Model components.

No.	Components	Description	Approximate half-life (hr)	Initial population size*
1	LPS	Lipopolysaccharide (endotoxin)	1.0	n/a
2	TLR4	Toll-like receptor 4	2.0	40
3	LPSR	LPS-TLR4 complex – active form	2.0	n/a
4	IKK	I kappa-B kinase complex – actived by LPSR	2.5	50
5	NFκB.IκB	NFκB complex – inactive form	2.5	50
6	NFκB	NFκB – active form	2.0	n/a
7	IκB	I kappa-B – NFκB inhibitors	0.5	10
8	P	Pro-inflammatory proteins – active when imported	1.5	30
9	A	Anti-inflammatory proteins– active when imported	1.5	30
10	E	Bio-energetic proteins	2.0	40
11	F	Cortisol– active when imported^$^	1.0	n/a
12	GR	Glucocorticoid receptors	2.0	40
13	FR	Cortisol-receptor complex – active form	2.0	n/a
14	M	Melatonin– active when imported	1.0	n/a

* the initial corresponding number of molecules within a cell; ^$^: the status of P, A, F, and M change to active when they are imported to the cytoplasm (cells) or brain compartment

### Agent rules and behaviors

Agents are simulated objects (cells, molecules) that follow specific instructions on how they behave and interact with other agents within or between compartments. The rule system is listed in **Table 2** and described briefly as follows. When LPS is recognized by its receptor TLR-4, a signal transduction cascade triggers downstream intracellular signalling modules to ultimately activate the transcription of inflammatory genes. Such transcriptional processes are assumed to be mainly regulated by transcription factors for which NFκB serves as a proxy whose activities, including activities of IKK and IκB in the NFκB-signaling module, have critical roles in the inflammatory response [64], [65]. Following the activation of NFκB through the phosphorylation of the inhibitor protein IκB by IKK, NFκB is translocated into the nucleus to activate the transcriptional processes resulting in the production of pro-inflammatory cytokines (e.g. TNFα) and IκB [66], [67], [68]. After released to the systemic circulation, these pro-inflammatory cytokines may bind to their corresponding receptors on the membrane of leukocytes and either further activate the NKκB-signaling module [67], [69] or lead to production of additional TLR-4 molecules [70], [71]. On the other hand, they also act as hormone-like signals that converge to activate the HPA axis to stimulate production of glucocorticoids [58], [59] or suppress nocturnal melatonin production [72], [73], [74]. While glucocorticoids have critical roles in the anti-inflammatory arm of the host defense system by inducing the expression of anti-inflammatory proteins such as IκB and anti-inflammatory cytokines (e.g. IL10) [58], [75], they also act as potential modulators that enhance the production of melatonin [73], [76], [77]. Melatonin, in turn, can modulate the production of pro-inflammatory cytokines [18], [61]. In addition, to establish the relationship between the inflammatory response and the cellular energetic state, we assumed that there are a number of bio-energetic molecules (E) in each cell which represent the overall cellular energetic status. If the number of bio-energetic molecules is positive, the cell can be able to produce new molecules; and if it does, the default production of bio-energetic molecules is inhibited. Since anti-inflammatory cytokines are responsible for the counter-regulation of the pro-inflammatory responses, it is hypothesized that they have a role in increasing the amount of bio-energetic proteins.

**Table 2:** Model rules.

No.	Rule definition
1	LPSR and P imported to cells from plasma can activate IKK; activated IKK can activate NFκB.IκB to NFκB
2	An individual NFκB in the nucleus has a probability of κp/κi to produce a new unit of P/IκB respectively
3	IκB inhibits NFκB activity by forming NFκB.IκB complex
4	P, A in the inactive form can be released to plasma if they lie on the membrane (boundary) of cells
5	P, A, F, M can be imported to cells from plasma if they hit a cell when moving in plasma
6	P, A, F, M after imported to cells from plasma will not be released to plasma again
7	An individual P in the nucleus has a probability of pt to produce a new unit of TLR4
8	An individual A in the nucleus has a probability of ae to produce a new unit of E
9	A inhibits P activity; both are degraded when they hit each other
10	An individual FR in the nucleus has a probability of fa/fi to produce a new unit of A/IκB respectively
11	NFκB activity in the nucleus is inhibited if the number of NFκB is less than the number of FR in the nucleus
12	FR inhibits the default system production of GR when in the nucleus
13	An individual F in the brain has a probability of fm to produce a new unit of M
14	An individual M in the nucleus has a probability of mp to produce a new unit of P
15	An individual P in the brain has a probability of pf to produce a new unit of F
16	P in the brain prevents F from producing M if the number of P is two folds more than that of F in the brain
17	NFκB, active P, FR, active M, and IκB can be translocated to the nucleus; they inhibit the default system production of E if they stimulate the nucleus activity to produce a new unit
18	A constant number of individuals F (C_fm_) are added with the probability of sin(0 ← π/2) for the time from 3:00AM to 9:00AM
19	A constant number of individuals M (C_fm_) are added with the probability of sin(0 ← π/2) for the time from 10:00PM to 2:00AM
20	Molecules are degraded after ∼t hr if there is no action except movements where t/2 is defined by the approximate half-life of molecular types

In our simulations, there are four types of compartments: the plasma, the brain, the cell cytoplasm, and the nucleus. The plasma contains the brain compartment and all simulated cells (50 in this study); each cell contains a cytoplasm and a nucleus. All agents move in a random fashion following the ‘random walk’ model on a 2-dimensional grid (see Materials and methods). The plasma and each cell have their own simulating grid while the brain and nucleus directly occupy a region in the plasma and corresponding cell simulating grid respectively. There is no special spatial arrangement for agents. However, there are a number of restrictions on which compartment a molecule can be in. Specifically, LPS can only move in the plasma compartment; LPSR, IKK, NFκB.IκB, E, and GR are only present in the cytoplasm; NFκB, IκB, and FR can be in both the cytoplasm and the nucleus; M and A cannot be in the brain compartment and F cannot be present in the nucleus while P can move between any compartment. TLR4 molecules after produced are transferred to the cell membrane i.e. when they reach the boundary of the corresponding cell simulating grid; they are fixed there until they are destroyed.

Molecules are translocated between compartments based on an import- and export procedure. In the plasma compartment, if a molecule has the same position with a cell or reach the region of the brain, the system will check to determine whether it is imported or not. Except LPS, other molecule types are imported to the brain and cells with the approximate probability of LPS-binding TLR4 to simulate the probability of the binding to receptors. This is approximately the initial number of TLR4 molecules in a cell divided by the number of positions on the boundary of the cell simulating grid, which is about 30%. For LPS molecules, a random position on the boundary of the cell simulating grid is assigned; if it is overlapped with the position of some TLR4 molecule, it will be imported. If imported to a cell, the molecule status is changed to ‘active’. In the cytoplasm compartment, active molecules are simply translocated to the nucleus compartment when they reach the nucleus regions in the corresponding simulating grid. On the other hand, when a molecule reaches the boundary of a compartment, it is exported to the outer compartment if it is not restricted (see **Materials S1** for details).

Each agent moves in a random direction for a random number of times with a random delay time for each movement. However, two interactive molecules 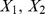 with current positions 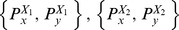 respectively will move towards the position where an interaction may occur if their distance is less than a threshold 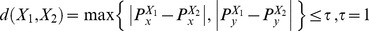. If two molecules have the same position on the simulating grid of the corresponding compartment, they will interact (activation, inhibition, or degradation) following the rules showed in **Figure 1a** and **Table 2** e.g. A and P with the same status in any compartment, LPSR and IKK, activated IKK and NFκB.IκB, F and GR in cytoplasm, and NFκB and IκB in cytoplasm or nucleus. The rule is also applied to the movement of molecules when adjacent to cells in the plasma to increase the probability of entering a cell for molecules in systemic circulation. Although there is no biological assumption in cell and molecule movements, these movement rules come from actual experiences in model implementation to increase the probability of agent interactions and the model performance.

Finally, circadian controls are introduced in an attempt to simulate the daily patterns of stress hormones [61], [62], [63]. In our simulation, these rhythms are produced using sine waves. At every tick during the time from the onset of the production to the corresponding peak in a day (e.g. 3:00AM to 9:00AM for cortisol and 10:00PM to 2:00AM for melatonin), a constant number of F and M units (c*_fm_*) are added to the system where the probability for each adding such a unit is
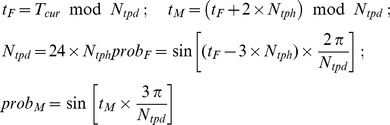



Simulated time is scaled from ‘ticks’, which is the simulation counter, to hours. 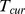 is the current tick of the simulation counter which expresses the current simulated time. 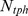 is the number of ticks corresponding to one simulated hour. c*_fm_* is selected to have the peaks of F and M approximately triple their corresponding homeostatic levels (c*_fm_* = 3 in this study). These activities are assumed to be controlled and taken place in the brain compartment since they are all associated with behaviors of the hypothalamus. Definition of the time-scale and the homeostatic system will be discussed in the following section.

### Model parameters

Model parameters are classified into two categories: default- and production- parameters. Default parameters are those related to system settings and physicochemical properties of cells and molecules, such as compartment extensions, simulation scales, molecule lifetimes, or initial populations. For simplicity, in this study all compartments are simulated with unitless rectangular grids. The plasma is represented by an 80 × 50 rectangular unitless grid and the cell with 40 × 30. The cell nucleus is about 10% of the total cell volume and thus it occupies a region of about 11 × 11 on the cell simulating grid. Similarly, the brain compartment is also simulated by a region of 11 × 11 on the plasma simulating grid (**Figure 1b**).

Since the relationship between the system response time and the system production rate is still unclear, we thus define two scales (an approximate number of simulated steps for an hour) in this simulation: (1) the life-scale 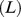 that characterizes for the lifetime of molecules and the system production rate, and (2) the time-scale 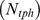 that characterizes for circadian controls and system responses. The time-scale is initially equal to the life-scale but adjusted later to match *in silico* system responses with *in vivo* transcriptional responses. In order to identify the life-scale, the system is set to have no activity except the default system production and the protein degradation; thus the number of units of each molecule type in a cell should be balanced over the time. Given the default production rate is 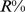, after an hour a cell will produce 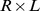 new units for a molecule type and thus there must be 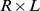 units of this molecule type degraded to keep the cell at homeostasis (*R* = 50% in this study). Consequently, if a molecule has a certain lifetime, its average lifetime will be approximately to its number of units divided by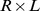. In other words, the initial number of units of a molecule type is set equally to its average lifetime multiplied by 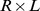.

In this simulation, the average lifetime of a unit is double its approximate half-life which is listed in **Table 1**. Specifically, IκB half-life is about 0.5 hour and the NFκB.IκB complex half-life is five-fold more than that of IκB [65], [78]; inflammatory cytokines and stress hormones have the average half-life about 1 hour [79], [80]; the largest protein IKK is assumed to have a half-life equal to that of the NFκB.IκB complex; and the rest are assumed to have the average half-life about 2 hours. Let f be the initial number of units of IκB in a cell, and the initial population of NFκB.IκB, IKK, P, A, E, TLR4, and GR in a cell will be (5f, 5f, 3f, 3f, 4f, 4f, 4f) respectively. Since intracellular protein occupies 15–35% of cell volume [81], we thus assume that the number of molecules in a homeostatic cell would be about 25% of the cell volume, which is approximately 300 molecules. Consequently, the total initial number of units (molecules) in a cell under the assumption of the homeostatic system will be 29f, resulting in 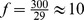 units. The estimated initial population size of each molecule type in a cell is given in **Table 1**. The life-scale , which is the number of simulated steps per hour or the number of simulated steps over the lifetime of an IκB, is therefore equal to f/R or 20 ticks per hour. Additionally, the initial number of units for P (or A) in the plasma is initialized by 10% of all P units in all cells in the system. The default production of F and M is set to be the activities of the brain compartment (see **Figure S1** for the programming architecture and initial parameter values).

Production parameters are the probabilities of producing new molecules when some molecule type is involved in the transcriptional process, characterizing by its present in the nucleus or the brain compartment in this simulation. In order to justify these parameters, we made use of the hypothesis that in a homeostatic system there is a balance between protein synthesis and protein degradation [82]. Thus, without any external stimulation and circadian influences, production parameters need to be adjusted so that the number of units of each molecule type in the system does not change significantly over the time (**Table 3**). Techniques from process trending analysis are utilized to obtain the set of adjusted parameters whose values remain unchanged for subsequently added mechanisms e.g. circadian rhythms, endotoxin treatments [83], [84] (see Materials and methods). The current configuration of the homeostatic system, including all agents and their properties, is saved for further experiments.

**Table 3:** Model production parameters.

No.	Parameters	Initial probability (%)	Adjusted probability (%)
1	κp (NFκB ← P)*	70.00	69.44
2	κi (NFκB ← IκB)	70.00	70.08
3	fi (F ← IκB)	70.00	70.08
4	fa (F ← A)	70.00	74.77
5	fm (F ← M)	70.00	27.48
6	mp (M ← P)	70.00	69.44
7	pf (P ← F)	70.00	24.98
8	pt (P ← TLR4)	70.00	70.00
9	ae (A ← E)	70.00	75.72

* x (Y Z): x is the probability that a single unit Y can produce an individual unit Z when Y is in the nucleus (or brain) compartment.

## Results and Discussion

### Qualitative assessment of model behaviors with experimental observations

**Figure 2:**
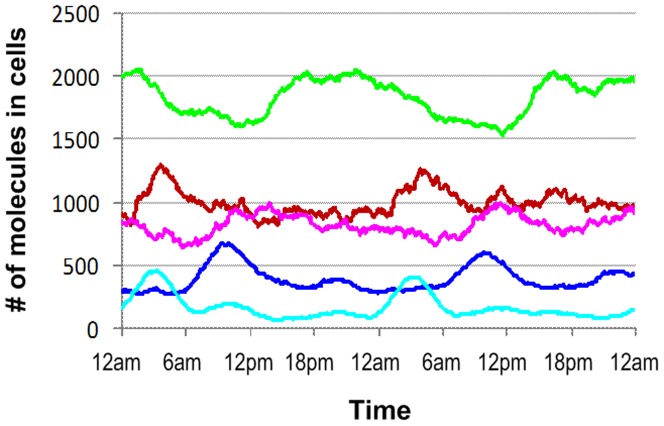
Dynamics patterns of selected components under circadian control. Circadian control is regulated by the rhythms of cortisol (F) and melatonin (M), which in turn drive the patterns of other components in the system. Pro-inflammatory cytokines (P), driven by melatonin secretion, are up-regulated to peak around ∼4:00AM whereas anti-inflammatory cytokines (A) are down regulated due to the increase of pro-inflammatory cytokines and then up-regulated under the effects of cortisol rhythms. These behaviors result in the circadian variation of bio-energetic proteins (E) and others.

Circadian rhythms play an important role in many physiological and metabolic processes in mammals. It is well established that there is a bidirectional communication between circadian controls and the immune system and that glucocorticoids and melatonin are important hormones with strong circadian expression patterns and critical roles in mediating cytokine production [60], [61], [62]. Since melatonin and cortisol are associated with the production of pro-inflammatory and anti-inflammatory cytokines respectively, their expression rhythms contribute to the dynamic patterns of cytokine expression [17], [18], [19], [62], resulting in the rhythms of P and A as observed in **Figure 2**.

Pro-inflammatory cytokines (e.g. TNFα, IL6, IFNγ) are regulated in part by melatonin, reaching a maximum in the early morning and subsequently subsiding as cortisols induce the production of anti-inflammatory cytokines (e.g. IL10). As pro- and anti-inflammatory cytokines have opposing effects on cellular immunity, changes in their concentration and thus their balance would be anticipated to influence the host fitness. Additionally, since transcription in the nucleus requires energy, each time a nucleus produces a new molecule besides the default system production, the corresponding cell will exhaust some unit of energy, representing by the deletion of one bio-energetic molecule (E) in this simulation. Consequently, energy balance and/or energetic protein abundance relevant to metabolic processes also exhibit daily circadian variations [85]. These observations provide a validation for our model’s behaviors.

*In silico* administration of endotoxin is simulated by ‘injecting’ a number of new LPS molecules into the system at tick *T* which is corresponding to time 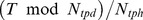 of the day. In order to simulate *in vivo* endotoxin administration at 9:00am, we introduce 1000 LPS molecules randomly to the plasma compartment at the corresponding tick and track the cellular responses. Due to lack of information to evaluate the corresponding dose and influence of other system factors (e.g. cell density) to the actual effects of those LPS molecules, we measure the effective concentration of LPS in our system by a definition as follows: 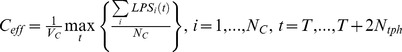. 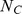 is the total number of simulated cells (50 in this study) and 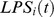 is the number of LPSR molecules in cell i at time t. 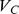 is the volume of cells which is equal to 30 × 40 = 1200 in this context. The effective concentration of this experiment is about 0.33%. The current default time-scale 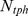 is 20 ticks per hour as discussed in the ‘Model parameters’ section. Since this time-scale calibration does not provide a corresponding mapping of the times between *in vivo* and *in silico* inflammatory responses, we vary 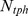 to search for a timing match between *in vivo* and *in silico* patterns by gradually increasing the number of ticks per hour to 30, 40, 50, etc. The search ends up with a new time-scale 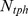 = 50.

**Figure 3:**
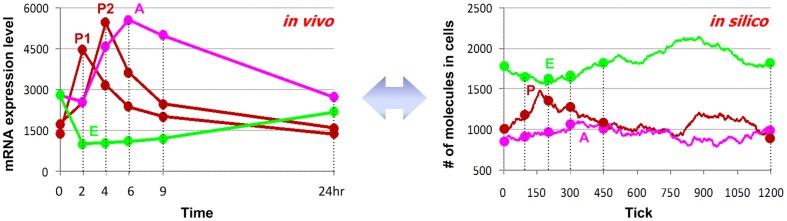
Correspondence between *in vivo-* and *in silico-* system responses to endotoxin. The left-panel presents average expression patterns of critical inflammatory responses under endotoxin treatment at 9:00AM. Early-up (red) and middle-up (black) patterns are characterized for pro-inflammatory responses, late-up pattern (magenta) for anti-inflammatory responses, and down pattern (green) for energetic responses. The right-panel displays corresponding simulated responses. The patterns between *in vivo-* and *in silico-* responses are matched to define the time-scale for the system.

The main inflammatory responses of *in vivo* and *in silico* human endotoxemia are presented in **Figure 3**. Following endotoxin treatment, the pro-inflammatory response exhibits a fast and robust increase, peaking between 2 and 4 hr after treatment and eventually resuming normal rhythms. The anti-inflammatory response, normally down-regulated around mid-day, keeps increasing following LPS administration. The systemic energy balance is also suppressed for around 2 hr more before returning to its normal rhythm (see more in **Figure S2**). The system resumes normal daily rhythms about 24 h post LPS administration.

### Patterns and implications of cellular variability

**Figure 4:**
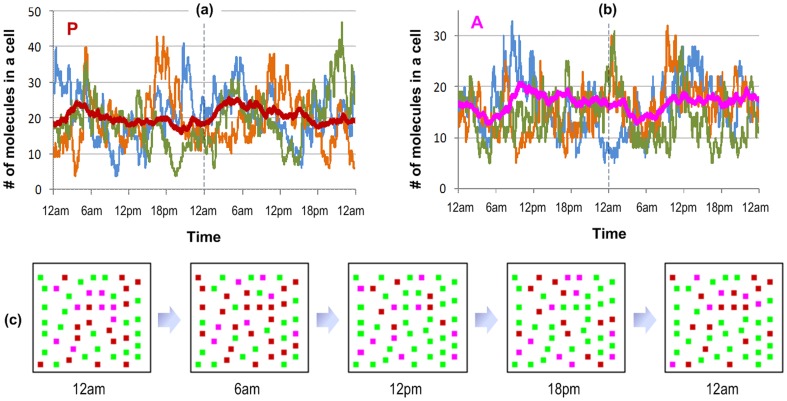
Stochastic dynamics in cell population. The stochastic behaviors of pro-inflammatory cytokines (a) and anti-inflammatory cytokines (b) in three different cells are shown in the top-panel. Although cellular patterns are different from cell to cell and from day to day, the average pattern still exhibits some daily common pattern. The dynamics of the homeostatic system in a simulated day are present in (c). Cells are displayed with solid squares where green squares represent for cells with an approximate number of P and A, red squares for those with the number of P much greater than the number of A and magenta squares for those with A >> P.

Since stochasticity is an inherent property of our individual-based simulation, stochastic transcriptional activities, especially those relevant to the NFκB-signaling module, have large impacts on cellular variability [86], [87], [88]. Simulated cells behave differently from one to another and no individual cell behaves like the average one. For example, dynamics patterns of pro- and anti-inflammatory protein levels oscillate stochastically between different cells and even different days although their average patterns exhibit some common daily patterns (**Figure 4a, b**). In general, these average patterns are similar to corresponding system responses. Specifically, the average level of pro-inflammatory cytokines is induced early due to the increasing level of melatonin at the onset of the day and then gradually abates while the level of cortisol increases. The average level of anti-inflammatory cytokines is transiently down regulated and then starts increasing to restore the balance between pro- and anti-inflammatory cytokines under the opposing effects and acutely altered patterns of melatonin and cortisol. From a system-level perspective, we assume that a cell will be (1) in the pro-inflammatory state (displayed by red squares) if the level of pro- is much greater than the level of anti-inflammatory cytokines (P>1.5A), (2) in the anti-inflammatory state (magenta squares) if A>1.5P, and (3) otherwise in the homeostatic state (green squares). Interestingly, the status change of the cellular system also follows a common daily pattern although the status of a single cell is always dynamic over time, even for the same time the next day (**Figure 4c**). At the beginning of a day, pro-inflammatory cells predominate and then make room for anti-inflammatory cells in the late morning. Since the status of the cellular system is in some part associated with the protein abundance level of corresponding cytokine types, the balance between pro- and anti-inflammatory cytokines is anticipated to be dynamic over time but follow some common daily pattern.

**Figure 5:**
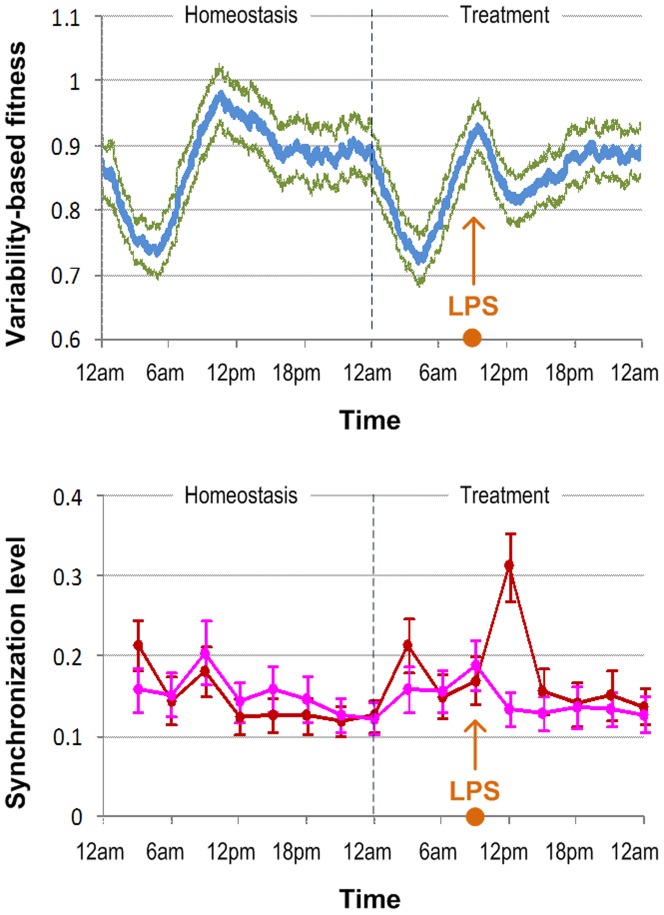
Cellular variability and synchronization behaviors. The top-panel displays the pattern of variability-based fitness of a simulated day in the homeostatic system and of the day where endotoxin is treated at 9:00AM. Two parallel curves present corresponding standard errors of N simulations (N = 100 in this study). The bottom panel shows the synchronization level of specific behaviors among all cells of the system in the interval [t – 3 hr, t], t = 3, 6…24 hr. The error bars are corresponding standard errors of N simulations.

Recent studies have implied that there is an association between patient fitness and the balance between the levels of pro- and anti-inflammatory cytokines [89], [90]. However, the protein abundance level in a population of genetically identical cells is proportional to the expression variance of the corresponding protein [91], [92], [93]. Consequently, the cell-to-cell variability potentially conveys information beyond the simple mean level of protein abundance in characterizing the dynamic kinetics of the entire system at the single cell level. Cellular variability can account for the stochastic transcriptional activities and thus not only the consequence but also the mechanisms that lead to the fluctuation of a protein between cells. As a result, we hereby define a novel quantity to characterize the entire status of the system in homeostasis or under treated conditions, so-called the variability-based fitness (F_var_), based on the ratio between the expression variance of anti-inflammatory cytokines and pro-inflammatory cytokines from the population of simulated leukocytes. In order to characterize the cytokine expression variance among cells, we utilize Shannon entropy to estimate the cellular variability based on the distribution of pro- or anti-inflammatory cytokines through the cell population (see Materials and methods). This measurement somewhat reflects change in the host fitness, since the anti-inflammatory arm characterizes for the ‘fitness’ restoration and the pro-inflammatory arm serves as the ‘fitness’ dysregulation. In homeostasis, the ratio is anticipated to remain at some optimal level while its normal rhythm has some daily common fluctuations in the first half of a day due to the circadian secretion of melatonin and cortisol (**Figure 5** **-top**). Following endotoxin treatment (at 9:00AM in this case), the variability-based fitness immediately reduces to the minimum point around 3-4 hr post injection and then gradually returns to the optimal level when the systemic manifestation of endotoxin abates, implying that the effect of endotoxin treatment can be quantified through this method.

Even in the presence of large variability in some molecule types within the population of cells, external stimulus signals (e.g. TNFα) can cause cell synchronization for a short period of time [86], [87]. The synchronization behavior of cellular responses is therefore examined to get an insight into how pro- and anti-inflammatory cytokines act under endotoxin treatments. Quantitatively, the synchronization level of a response (e.g. a molecule type) is defined as the average correlation coefficient between all individual response patterns of cells and the average response pattern of the cell population in a period of time (e.g. 3 hr in this study) (see Materials and methods). LPS-induced cell synchronization has been examined for pro- and anti-inflammatory responses (**Figure 5** **-bottom**). Although the cellular pro-inflammatory responses are different from cell to cell, under an external stimulus their responses become more similar in the first time period directly after LPS treatment. However, anti-inflammatory responses among cells do not lead to a significant trend of synchronization. This phenomenon results from the fact that all cells follow the only path that activates the NFκB-signaling module to produce pro-inflammatory cytokines under the primary stimulus signal, while the path to produce anti-inflammatory cytokines is secondary and set under the effects of pro-inflammatory inhibitors. After the first period, stochastic oscillations resume in the population of cells although the systemic manifestation of inflammation does not quite abate.

### Time-dependent effects under endotoxin treatment

**Figure 6:**
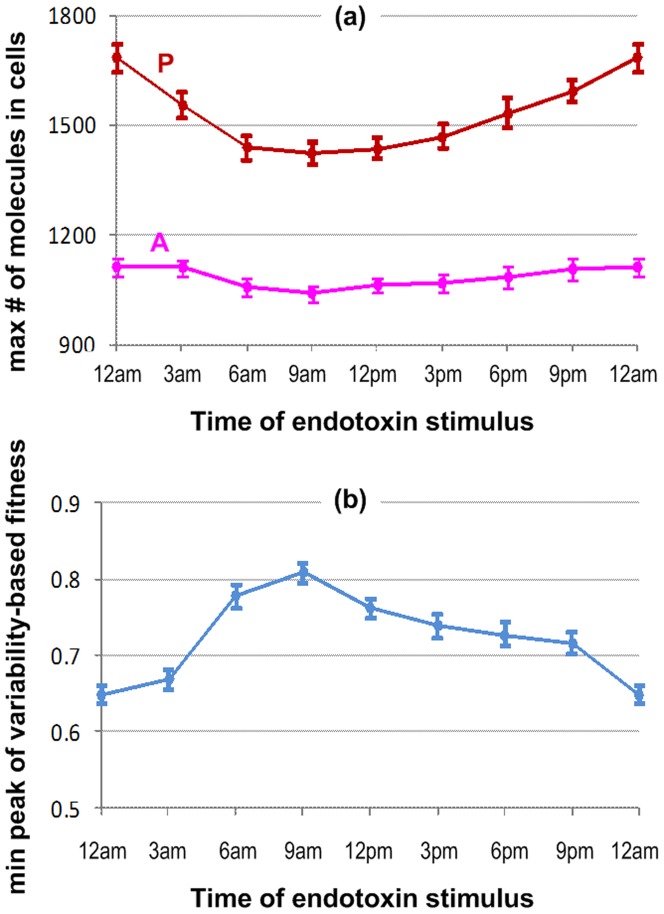
Time-dependence system responses to endotoxin administration. The strength of the inflammatory response or the vulnerability of the host fitness is characterized by (a) the maximal peak of pro-inflammatory cytokines (P_max_) and (b) the minimum peak of variability-based fitness versus the time of endotoxin treatment. The error bars are corresponding standard errors of N simulations (N = 100).

As observed in previous studies, there are clearly significant effects of circadian rhythms on the dosing time in therapeutic treatments. For instance, “low dose prednisolone has more effect on rheumatoid arthritis at 2:00AM than at 7:00AM” [17], [19] and “bedtime dosing with nifedipine gastrointestinal therapeutic system for antihypertensive medications is more effective than morning dosing” [94], [95]. We therefore explore the time-dependent effects of endotoxin administration by executing *in silico* experiments with endotoxin injection at different times of the day (3 hr intervals from 0 to 24 hr). We quantitatively examined the peaks of inflammatory responses following endotoxin administration at different times throughout the day. Results are characterized by the maximum numbers of pro- and anti-inflammatory cytokines as well as the peak of the variability-based fitness versus the treated times of endotoxin (**Figure 6**) (see more details in **Figure S2**). Simulation shows that endotoxin administrated in the morning (around 9:00AM) has the least pronounced effect, while the largest response occurs around midnight. Although the maximum numbers of anti-inflammatory cytokines in different cases seem to be approximately equal, there is a significant trend in the effects of administration times of endotoxin on the production of pro-inflammatory cytokines. Characterizing these phenomena is the change of the variability-based fitness versus the administration time, implying somehow the loss of the host fitness. Periods of highly vulnerable effects are those around the midnight peak of melatonin secretion where the production of pro-inflammatory cytokines is regulated by two pathways, NFκB-signaling and the melatonin-induced pathway. On the contrary, high concentration of plasma cortisol in the morning provides an inhibitory effect on the activation of the NFκB-signaling module, resulting in reduced effects of endotoxin administration.

### Sensitivity analysis

Sensitivity analysis was performed to explore how perturbations in production parameter values affect the overall system behavior, as characterized by the variability-based fitness. Following previous studies [25], [50], we sequentially perturbed each production parameter and estimated the sensitivity coefficient which is defined as the percentage change of the fitness (DF_var_) over the percentage change of the parameter (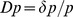 where 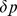 is the changing amount of parameter p) (see Materials and methods). In this case, 75% is selected as the cutoff to have a clear impact on the percentage change of the fitness, which is estimated from 10 simulated days with circadian controls and no external stimulus. Results are showed in **Table 4**. The two parameters that have great impact are κi and fa respectively, where κi is responsible for IκB production from the NFκB activity and fa is directly responsible for the production of anti-inflammatory cytokines. Since the sensitivity coefficient is mainly dependent on the change of the variability-based fitness where the dynamics of pro- and anti-inflammatory cytokines take place, parameters relevant to the production of these cytokines should have large impact. However, since κi affects IκB production from NFκB activity in the nucleus which in turn directly control back NFκB activity in regulating the production of pro-inflammatory cytokines, a small change on the value of κi can have a large impact on the regulation of pro-inflammatory cytokine production. Therefore, κp and mp, two parameters directly relevant to the production of pro-inflammatory cytokines, have lesser impacts on the variability-based fitness than κi does.

**Table 4:** Effects of production parameters on system behaviors.

No.	Parameters	Change*	DF_var_/Dp	Change*	DF_var_/Dp
1	κp	↓	0.2085	↑	0.2461
2	κi	↓	0.4481	↑	0.2679
3	fi	↓	0.1861	↑	0.2520
4	fa	↓	0.3260	↑	0.3485
5	fm	↓	0.1560	↑	0.1440
6	mp	↓	0.1883	↑	0.2452
7	pf	↓	0.1607	↑	0.1585
8	pt	↓	0.1391	↑	0.1429
9	ae	↓	0.1425	↑	0.3303

* decrease/increase 75% of the current value; if greater than 1.0, set to 1.0.

In summary, we have proposed a multi-level homeostatic system of human endotoxemia using the individual-based simulation. The model is used to examine the dynamic kinetics of the inflammatory response at the single cell level under circadian control and endotoxin treatment. Physicochemical properties of biological molecules and cellular properties have been incorporated to construct the model. Novel solutions for parameter tuning and time-scale estimation are also proposed to refine the parameters. Although the model is able to reproduce *in vivo* homeostatic circadian rhythms and key inflammatory responses under endotoxin treatment, limitations still remain. One of the main issues is the proper identification of parameters associated with the simulated compartments (e.g. compartment dimension, cell density) and the import/export rate. Also, we have to accept a level of abstraction in our model where only key components and behaviors in the signaling cascade and the transcriptional process are captured while ignoring a lot of biological processes. There is still lack of understanding of mechanisms relevant to melatonin and cortisol activities. More importantly, there are a variety of leukocytes with multiple pro- and anti-inflammatory cytokines. Each may have different regulatory mechanisms but in this study we assumed they act similarly. Finally, another weakness is that our current approach relates to our limited ability to quantify the magnitude of the inflammatory responses as well as other marker levels. These limitations present key targets in our future studies.

However, one of the most critical questions raised in this study is to what extent cellular variability can contribute to systemic outcomes. By defining novel hypothetical quantities such as the variability-based fitness and the synchronization level, we provided a step forward to the exploration of cell-to-cell variability and stochastic dynamics of inflammatory proteins. Daily common patterns of such measurements in homeostatic and LPS-treated systems are examined. Furthermore, the effects of time-dependent endotoxin administration characterized by the variability-based fitness and the synchronization level of inflammatory cytokines are also studied. Although a full understanding of how cell-to-cell variability impacts clinical symptoms and pharmacological treatments is beyond the scope of this manuscript, proposed concepts in this study may actually be applicable in the near future as single-cell studies become increasingly common. Also, the proposed framework provides an effective model to generate testable hypotheses for a number of ‘*what if*’ scenarios to understand the connectivity of critical components in the immune system and the interplay between circadian controls and endotoxin treatments.

## Materials and Methods

### Human endotoxin model and data collection

The data used in this study were generated as part of the Inflammation and Host Response to Injury Large Scale Collaborative Project funded by the USPHS, U54 GM621119 [96]. Human subjects were injected intravenously with endotoxin (CC-RE, lot 2) at a dose of 2-ng/kg body weight (endotoxin treated subjects) or 0.9% sodium chloride (placebo treated subjects). Following lysis of erythrocytes and isolation of total RNA from leukocyte pellets, biotin-labeled cRNA was hybridized to the Hu133A and Hu133B arrays containing a total of 44,924 probes for measuring the expression level of genes that can be either activated or repressed in response to endotoxin. Subsequently, ANOVA test (p<10^-4^) was applied to filter significantly differentially expressed probesets, resulting in 3,269 selected probesets [46]. Average expression profiles of probesets over replicates for each time-point were used as the final input data for further analyses. The data are publicly available through the GEO Omnibus Database under the accession number GSE3284. The data have been appropriately de-identified, and appropriate IRB approval and informed, written consent were obtained by the glue grant investigators [96].

#### Model implementation

The *in silico* human endotoxemia model is implemented in Java language, using the Repast Simphony toolkit and Eclipse environment.

### Clustering

Utilizing the concept of the agreement matrix (AM) in consensus clustering, we recently proposed a novel method to identify the core set of probesets showing most agreeable that they belong to the same or different patterns of gene expression [46]. In order to produce the agreement matrix, a number of different clustering methods along with different metrics (Euclidean, Manhattan, and Pearson correlation) were used to reduce the bias inherent in the assumption of any specific clustering method. After identifying the core set of probesets, the AM is reduced correspondingly to those selected probesets and then the hierarchical clustering is applied on the reduced AM to obtain significant patterns of gene expression. Four patterns of gene expression which characterize critical dynamics of acute human inflammation are obtained [46]. The ‘early-up’ (182 probesets) and ‘middle-up’ pattern (119 probesets) consist of genes that are involved in critical pro-inflammatory signaling pathways including apoptosis, Toll-like receptor (TLR) signaling, and cytokine-cytokine receptor interaction. The ‘late-up’ pattern (284 probesets) characterizes for anti-inflammatory processes with enriched inflammatory relevant pathways e.g. TLR signalling, JAK-STAT cascade. Finally, the ‘down’ pattern (1118 probesets) is the most populated expression motif characterized by genes involved in cellular bio-energetic processes e.g. oxidative phosphorylation, ribosome, TCA cycle, purine and pyruvate metabolism.

### ‘Random walk’ model

Agents (cells, molecules) move on a 2-dimensional grid in a random fashion depending on two main factors: the time agents wait before each movement and the number of times agents move in a direction. For a specific agent U, at time t, let 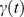 be the time (number of ticks) U has to wait before moving and  be the number of times U will move in direction D, we have
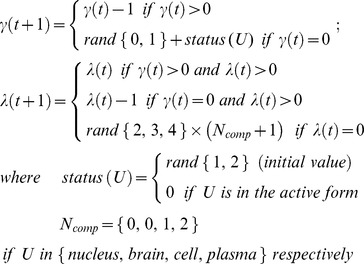



Each compartment or each cell has its own 2-dimensional simulating grid. When 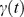 is zero, U will move to the next grid-space in the Moore neighbourhood of the corresponding simulating grid which consists of 8 spaces immediately adjacent to and surrounding the current position based on the current direction D. D is one of 8 directions 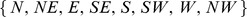 (N: north, E: east, S: south, and W: west). Let 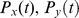 be the current position of U in a 2-dimensional simulating grid, its next position is defined as follows
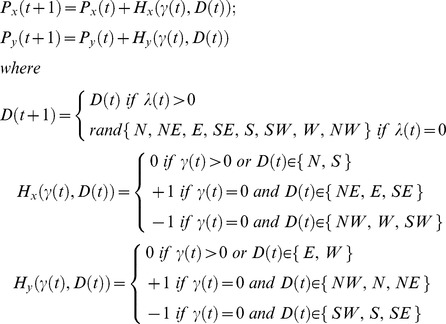



### Parameter tuning

Based on the trend of the dynamics of each particular molecule type 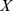, we adjust the probability of the associated production parameter 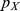 (**Table 3**) so that the total number of 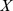 in the system does not change significantly over time. For each simulated day (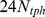 ticks), we sample the level of X each hour and determine whether there is a significant change based on the sample vector using ordinary least square regression and significant mean difference [83].

Let 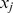 be the number of molecules 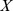 in the system at hour 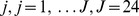. The regression model used in this approach is 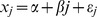 where 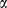 is the intercept, 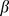 is the slope, and 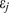 are random errors which are assumed to be independent and identically distributed. The estimates of the slope and intercept are given by
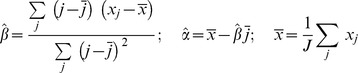



The standard error of the slope will be 
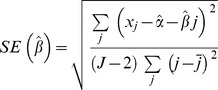


A 95% confidence interval for the slope 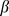 is 
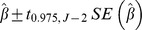
. If zero is not contained in the interval, we conclude that the trend of change is significant.

Let 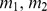 be the means of the first and last half of the sample vector
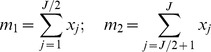



If the percentage change between the first and last half of the sample vector 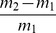 is more than 10%, we conclude that the change is significant and adjust the corresponding production parameter. If the trend of the dynamics is increasing, the parameter value 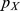 will be decreased. Otherwise, if the trend of the dynamics is decreasing, we increase 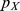. In order to estimate the changing amount of 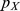, we assume that the percentage change of the parameter would be approximately to the percentage change of the molecule level between the first and last half of the sample vector but set under the opposite effect. Therefore, the estimate for the adjusted parameter value will be
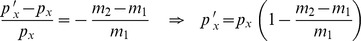



In the case that there are two associated production parameters, the amount each parameter is changed will be half of that in the normal case. The process is repeated until there is no change of all production parameters in three consecutive simulated days.

### Variability-based fitness

Given 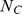 cells, let 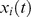 be the number of molecules 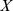 in cell 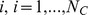 at a specific time 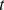. Since the distribution of 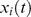 values may be sparse, we first contract the range of 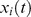 by a whole number division of 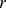 for all 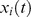 (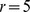 in this study). 
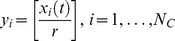



Let 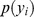 be the probability of the presence of 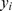 value in the contracted array 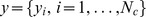. The variability-based fitness 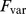 at a specific time 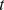 is defined as follows
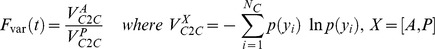



### Synchronization

Let 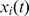 be the number of molecules 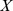 in cell 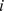 and 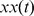 be the average number of molecules 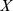 from 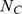 cells at time 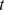. The synchronization level of molecule 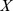 in the population of cells for a period of time from 0:00 to 3:00AM is
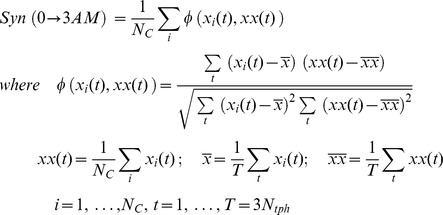



### Percentage change of the fitness

In order to evaluate how a change impacts to the system behaviors, we define a so-called percentage change of the fitness as a ratio of the total changing amount between the variability-based fitness of the original system and that of the new system over the total amount in the original system during a period of time (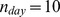 in this study)
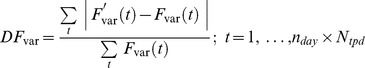



where 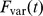 and 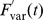 are the variability-based finesses at time t of the original system and the new system (i.e. the system with new parameter values).

## Supporting Information

Figure S1 **Model architecture of the programming.** There are two general classes (*SimpleAgent* and *SimpleCompartment*) which are inherited by other classes in the system. Connecting lines ending with the empty triangle reflect the inheritance between the general class and a specific class while those ending with the solid parallelogram exhibit an aggregation of containing relationships. Initial parameters are also showed in the right table.
(TIFF)

Figure S2 **System responses under a bolus injection of endotoxin at different times of the day.** Each case includes the average system behaviors of inflammatory cytokines and stress hormones from N simulations (N = 100 in this study), the corresponding pattern of variability-based fitness, and the synchronization level of pro- and anti-inflammatory responses following intervals [t – 3 hr, t], t = 3, 6…24 hr. The error bars (or two parallel curves) are corresponding standard errors of N simulations.
(TIFF)

Materials S1 **Pseudo-code of the implemented model.**
(DOC)
