# Supplementary figures and images for: An Agent-Based Model of Cellular Dynamics and Circadian Variability in Human Endotoxemia
Source: PLoS One. 2013 Jan 30;8(1):e55550. doi: 10.1371/journal.pone.0055550 (PMC3559552; doi:10.1371/journal.pone.0055550)

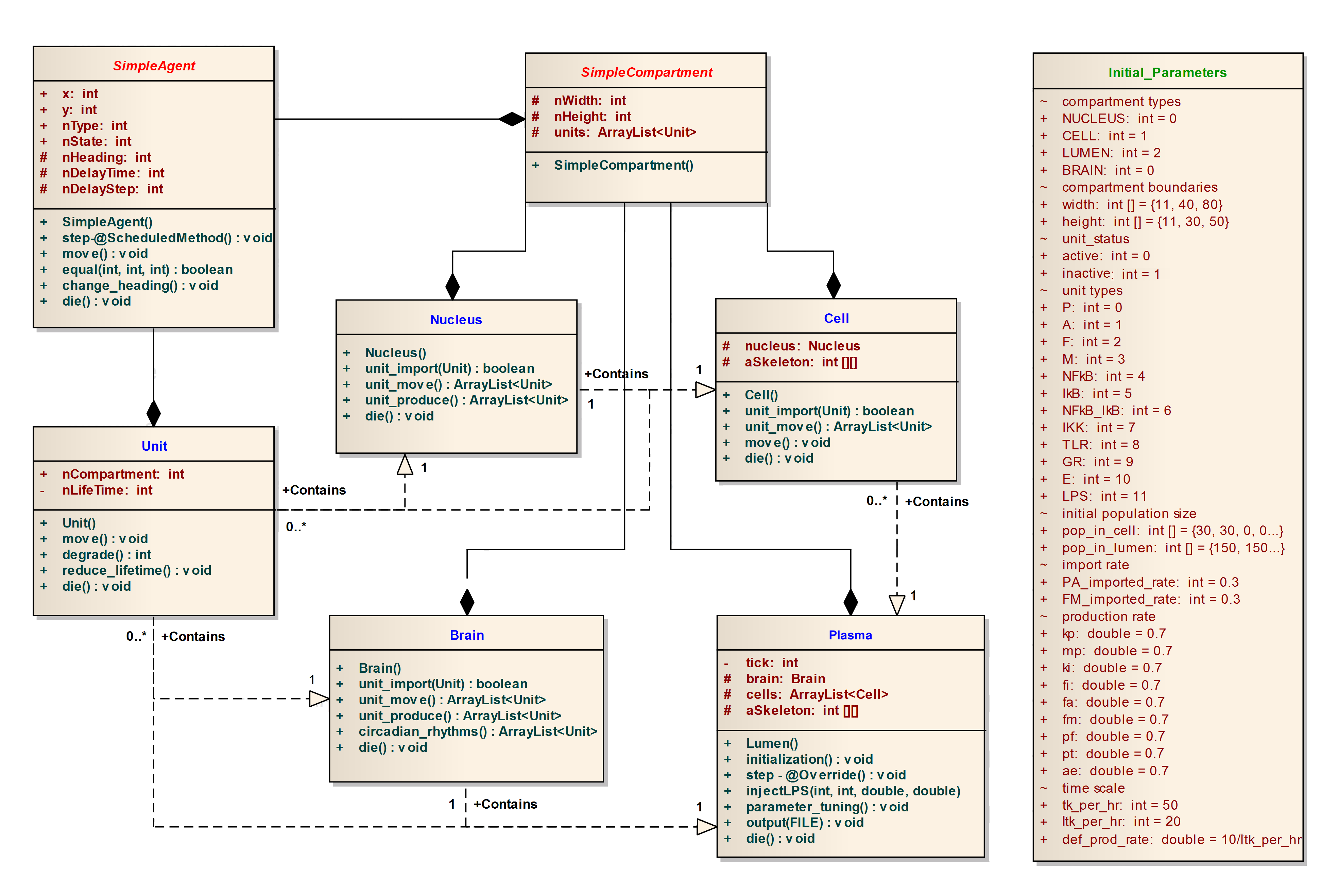

Supplement: Figure S1 — Model architecture of the programming. There are two general classes (SimpleAgent and SimpleCompartment) which are inherited by other classes in the system. Connecting lines ending with the empty triangle reflect the inheritance between the general class and a specific class while those ending with the solid parallelogram exhibit an aggregation of containing relationships. Initial parameters are also showed in the right table. (TIFF) [file pone.0055550.s001.tiff]

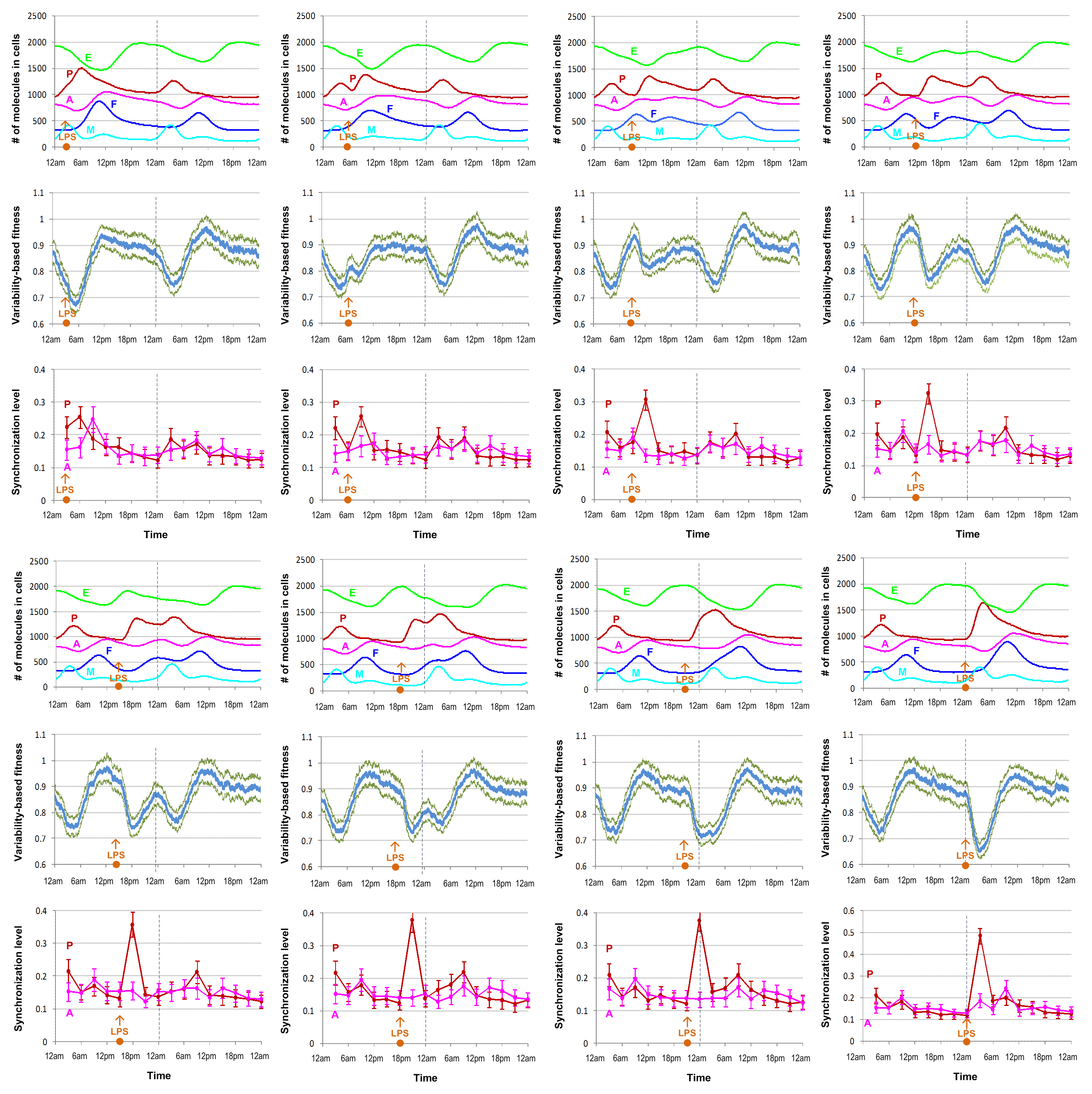

Supplement: Figure S2 — System responses under a bolus injection of endotoxin at different times of the day. Each case includes the average system behaviors of inflammatory cytokines and stress hormones from N simulations (N = 100 in this study), the corresponding pattern of variability-based fitness, and the synchronization level of pro- and anti-inflammatory responses following intervals [t – 3 hr, t], t = 3, 6…24 hr. The error bars (or two parallel curves) are corresponding standard errors of N simulations. (TIFF) [file pone.0055550.s002.tiff]
