# Supplementary material for: An Agent-Based Model of Cellular Dynamics and Circadian Variability in Human Endotoxemia
Source: PLoS One. 2013 Jan 30;8(1):e55550. doi: 10.1371/journal.pone.0055550 (PMC3559552; doi:10.1371/journal.pone.0055550)
Supplement: Materials S1 — Pseudo-code of the implemented model. (DOC) [file pone.0055550.s003.doc]

**Supplemental Materials 3**

Pseudocode of the human endotoxemia ABM

////////////////////////////////////////////////////////////////////////////////

// Activities of a molecule

//

// initialization

molecule type

molecule state

molecule lifetime

random moving direction (heading)

random coordination (x,y) in the corresponding compartment

// activities

// 1. molecule moving, time-delayed and compartment-restricted

if delaytime>0

delaytime <- -1

return

else

delaytime <- rand{1,2,3}

if the state is "activated"

delaytime <- rand{0,1}

end if

end if

switch (heading)

case N: y++; break;

case NE: x++; y++; break;

case E: x++; break;

case SE: x++; y--; break;

case S: y--; break;

case SW: x--; y--; break;

case W: x--; break;

case NW: x--; y++; break;

end switch

check the heading change and reassign it following the model in the manuscript

// 2. molecule degradation

lifetime <- -1

if lifetime<0

send a signal to destroy it

end if

////////////////////////////////////////////////////////////////////////////////

// Activities of the nucleus in a cell

//

// initialization

nucleus dimension

fixed coordination in the cell space

// activities

// 1. import molecules from corresponding cell cytoplasm if possible

if the molecule is **NFKB**, **IKB** or (**P**, **A**, **FR**, **M** and "activated")

create a duplicate of the molecule with a random coordination in the nucleus compartment

send a signal to accept the import

end if

// 2. nucleus operations e.g. molecule moving, interactions and export if any

for each molecule in the nucleus

molecule degradation

randomly moving followed the molecule moving function

end for

for each molecule in the nucleus

if the molecule position is on the nucleus boundary

create a duplicate of the molecule with new random coordination in cytoplasm

send it to the corresponding cytoplasm

send a signal to destroy it

end if

end for

for each pair of molecules in the nucleus

if they have the same coordination

if the molecules are **A** and **P**

send a signal to destroy both

end if

if the molecules are **NFKB** and **IKB**

create a new complex **NFKB**.**IKB** in the corresponding cytoplasm

send a signal to destroy both

end if

end if

end for

// 3. produce new molecules due to transcription activities

for each molecule in the current nucleus

prob <- rand(0,1)

if the molecule is **P** and prob<**pt**

produce a new **TLR4** in the corresponding cytoplasm

end if

if the molecule is **A** and prob<**ae**

produce a new **E** in the corresponding cytoplasm

end if

if the molecule is **M** and prob<**mp**

produce a new **P** in the corresponding cytoplasm

end if

if the molecule is **NFKB**

if **NFKB**'s concentration is greater than **FR**'s concentration

prob <- rand(0,1)

if prob<**kp**

produce a new **P** in the corresponding cytoplasm

end if

prob <- rand(0,1)

if prob<**ki**

produce a new **IKB** in the corresponding cytoplasm

end if

end if

end if

if the molecule is **FR**

if **FR**'s concentration is greater than **NFKB**'s concentration

prob <- rand(0,1)

if prob<**fa**

produce a new **A** in the corresponding cytoplasm

end if

prob <- rand(0,1)

if prob<**fi**

produce a new **IKB** in the corresponding cytoplasm

end if

end if

end if

end for

////////////////////////////////////////////////////////////////////////////////

// Activities in the cytoplasm of a cell

//

// initialization

cell dimension

initialized molecules

initialized nucleus

random moving direction (heading)

random coordination (x,y) in the plasma

// activities

// 1. cell moving, time-delayed and randomly

if delaytime>0

delaytime <- -1

return

else

delaytime <- rand{4,5,6}

end if

position (x,y) changed depended-on the heading

heading changed if possible, following the model in the text

// 2. import molecules from plasma to cell cytoplasm

prob <- rand(0,1)

if the molecule is **A** or **P** and prob<PA_imported_rate

send a signal to accept the import

create a duplicate of the molecule with a random coordination in the cytoplasm

change its state to "activated"

end if

if the molecule is **F** or **M** and prob<FM_imported_rate

send a signal to accept the import

create a duplicate of the molecule with a random coordination in the cytoplasm

change its state to "activated"

end if

if the molecule is **LPS**

generate a random position on the cytoplasm boundary

if this position is occupied by a **TLR4**

send a signal to accept the import

create a duplicate with a random coordination in the cytoplasm

end if

end if

// 3. cell operations e.g. molecule moving, interactions, and export if any

// molecule degradation and moving

for each molecule in the cytoplasm

molecule degradation

randomly moving followed the molecule moving function

if its interactive molecule is on the adjacent region (8 adjacent positions)

they are moved to the same position (x,y)

end if

end for

// export molecules to the plasma

for each molecule in the cytoplasm

if it is on the cell boundary

if the molecule is **A** or **P** and its state is not "activated"

create a duplicate of the molecule with new random coordination in plasma

send it to the plasma

send a signal to destroy it

else

change its heading

end if

end if

end for

// import molecules to the nucleus if possible

for each molecule in the cytoplasm

if it is on the nucleus boundary

check with the nucleus

if it can be imported to the nucleus

ask the nucleus to import the molecule

send a signal to destroy it

else

change its heading

end if

end if

end for

// molecule interactions

for each pair of molecules in the cytoplasm

if they have the same coordination

if the molecules are **IKK** and (**LPSR** or **P** with the "activated" state)

change the state of **IKK** to "activated"

send a signal to destroy **LPS** or **P**

end if

if the molecules are "activated" **IKK** and complex **NFKB**.**IKB**

create a new molecule **NFKB** in the corresponding cytoplasm

change its state to "activated"

change the state of **IKK** to "inactivated"

send a signal to destroy **NFKB**.**IKB**

end if

if the molecules are **NFKB** and **IKB**

create a new complex **NFKB**.**IKB** in the corresponding cytoplasm

send a signal to destroy both

end if

if the molecules are **F** and **GR**

create a new molecule **FR** in the corresponding cytoplasm

change its state to "activated"

send a signal to destroy both **F** and **GR**

end if

if the molecules are **A** and **P** and they have the same state

send a signal to destroy both

end if

end if

end for

// synchronize nucleus activities

ask the nucleus to perform nucleus operations and transcription activities

////////////////////////////////////////////////////////////////////////////////

// Activities in the brain

//

// initialization

brain dimension

fixed random coordination in the system

// activities

// 1. import molecules if possible

prob <- rand(0,1)

if the molecule is **P** and prob<PA_imported_rate

create a duplicate of the molecule with a random coordination in the brain compartment

send a signal to accept the import

end if

if the molecule is **F** and prob<FM_imported_rate

create a duplicate of the molecule with a random coordination in the brain compartment

send a signal to accept the import

end if

// 2. molecule moving

for each molecule in the brain compartment

molecule degradation

randomly moving followed the molecule moving function

if it is on the brain boundary

create a duplicate of the molecule with new random coordination in plasma

send it to the plasma

send a signal to destroy it

end if

end for

// 3. produce new molecules due to **HPA** activities

for each molecule in the brain compartment

prob <- rand(0,1)

if the molecule is **P** and prob<**pf**

produce a new **F** with random coordination in the plasma compartment

end if

if the molecule is **F** and prob<**fm**

produce a new **M** with random coordination in the plasma compartment

end if

end for

// 4. produce new molecules to simulate the circadian rhythmicity

get the simulated system zeitgeber time (time <- current_simulated_ticks mod **Ntpd**)

if the time is between 3:00AM and 9:00AM

num_of_added_F <- 3

probF <- sin((time-3***Ntph**)*2***PI** / **Ntpd**);

for i from 1 to num_of_added_F

prob <- rand(0,1)

if prob < probF

produce a new **F** with random coordination in the plasma compartment

end if

end for

end if

if the time is between 10:00PM and 2:00AM

num_of_added_M <- 3

probM <- sin((time+2***Ntph**)*3***PI** / **Ntpd**);

for i from 1 to num_of_added_M

prob <- rand(0,1)

if prob < probM

produce a new **M** with random coordination in the plasma compartment

end if

end for

end if

////////////////////////////////////////////////////////////////////////////////

// Activities in the plasma

//

// initialization

plasma dimension

initialized brain compartment

initialized number of cells

initialized number of molecules in plasma

// Scheduler starts at tick one with an interval of 1 tick

// -> recurs at 2, 3, 4..., etc.

// ticks control and synchronize the entire system activities

// @Override public void step()

current_simulated_ticks <- +1

// cell moving

for each cell in the plasma

randomly moving followed the cell moving function

if it is on the plasma boundary or the brain boundary

change its heading

end if

if there is another cell with the same coordination

change its heading

perform one more randomly moving

end if

end for

// molecule moving

for each molecule in the plasma

molecule degradation

randomly moving followed the molecule moving function

if it is on the brain boundary

check with the brain compartment

if it can be imported to the brain compartment

ask the brain compartment to import the molecule

send a signal to destroy it

else

change its heading

end if

if it has the same coordination with a cell

check with that cell

if it can be imported to that cell cytoplasm

ask the cell to import the molecule

send a signal to destroy it

else

change its heading

end if

end if

end for

// molecule interactions

for each pair of molecules in the plasma

if they have the same coordination

if the molecules are **A** and **P**

send a signal to destroy both

end if

end if

end for

// synchronize all cells and brain activities

ask the brain to perform its functions

e.g. molecule moving, **HPA** activities, circadian rhythmicity

for each cell in the plasma

ask the cell to perform cell operations

end for
